# Selective Sphingosine 1-Phosphate Receptor 1 Modulation Augments Thrombolysis of Low-Dose Tissue Plasminogen Activator Following Cerebrovascular Thrombosis

**DOI:** 10.3389/fimmu.2022.801727

**Published:** 2022-05-27

**Authors:** Han-Dong Li, Ran Li, Ying Kong, Wenyan Zhang, Caiyun Qi, Dan Wang, Hongying Hao, Qiang Liu

**Affiliations:** Department of Neurology, Institute of Neuroimmunology, Tianjin Medical University General Hospital, Tianjin, China

**Keywords:** two-photon microscopy, tPA, thrombolysis, microcirculation, ischemic stroke

## Abstract

**Background:**

Results from our recent study demonstrate that sphingosine-1-phosphate receptor 1 (S1PR1) modulation improves microvascular hemodynamics after cerebrovascular thrombosis. This study was to determine the microvascular hemodynamic effects of a sub-thrombolytic dose of tPA combined with a selective S1PR1 modulator ozanimod in a mouse model of cerebrovascular thrombosis.

**Methods:**

Microvascular circulation in mice was monitored *in vivo* by two-photon microscopy. Thrombosis was induced in cortical arterioles by laser irradiation. Arteriolar flow velocity was measured by line-scanning following plasma-labeling with fluorescein-dextran.

**Results:**

Laser‐induced thrombosis led to a persistent reduction of flow velocity in cortical arterioles. Sub-thrombolytic dose of tPA along with vehicle control did not improve the flow velocity in cortical arterioles following thrombosis. In contrast, a sub-thrombolytic dose of tPA along with ozanimod dramatically restored flow velocity in cortical arterioles following thrombosis. Ozanimod did not affect coagulation and bleeding time. Notably, ozanimod reduced thrombus volume without altering microvascular lumen diameter. In addition, ozanimod reduced leukocyte components within the thrombus.

**Conclusions:**

These findings demonstrate that the thrombolytic effect of a sub-thrombolytic dose of tPA is markedly enhanced by S1PR1 modulation, implying that S1PR1 modulation may improve the therapeutic benefit of low-dose tPA in patients with acute ischemic stroke.

## Introduction

Thrombolysis with tissue-type plasminogen activator (tPA) is the only FDA-approved pharmacological treatment of acute ischemic stroke (AIS), despite the increased risk of intracerebral hemorrhage. Although low-dose tPA thrombolysis reduces the risk of hemorrhage, the mortality and disability rates are increased in AIS patients receiving a lower dose of tPA (1–4), presenting an unmet medical need for adjunctive therapies that can enhance the thrombolytic effects of low-dose tPA and maintain its safety advantage.

Sphingosine-1-phosphate receptor (S1PR) modulation provides protection in preclinical and clinical studies for ischemic stroke (5–7). Fingolimod is a S1PR modulator that inhibits the transmigration of lymphocytes from peripheral lymphoid organs into the neurovascular unit, and thereby reduces circulating lymphocytes within the cerebrovascular compartment, leading to improved collateral flow and barrier integrity (8, 9). Evidence has demonstrated the efficacy of selectively targeting S1PR1 to attenuate ischemic brain injury, indicating that the benefit of fingolimod mainly results from its action on S1PR1 (10–12). In a recent study using two-photon laser-scanning microscopy *in vivo*, we reported that selective S1PR1 modulation improves microvascular circulation after cerebrovascular thrombosis (13), suggesting that the efficacy of thrombolytic therapy in AIS may be enhanced by S1PR1 modulation.

Ozanimod is a selective S1PR1 modulator with >200-fold selectivity for S1PR1 over S1PR5, and >20,000-fold selectivity over S1PR2-4 (14). While reperfusion therapy using tPA is effective for occlusive stroke, the short therapeutic window and risk of hemorrhage limit the benefit of tPA in patients with ischemic stroke, highlighting an urgent yet unmet need for a combination treatment that would reduce tPA-associated hemorrhagic transformation to extend its efficacy and therapeutic window. Therefore, this study explored the impact of a selective S1PR1 modulator ozanimod combined with sub-thrombolytic dose of tPA on microvascular hemodynamics using a mouse model of laser-induced cerebrovascular thrombosis.

## Material and Methods

### Mice

Male and female C57BL/6 mice, 8-10 weeks old, were purchased from SPF Biotechnology Co., Ltd. (Beijing, China). Mice were maintained in pathogen-free conditions with standardized light-dark cycle conditions and free access to food and water. Animals were randomly assigned to each treatment group using QuickCalcs (GraphPad Software, La Jolla, CA, USA). All procedures were performed in accordance with the National Institutes of Health Guide for the Care and Use of Laboratory Animals and were approved by Animal Care and Use Committees of Tianjin Neurological Institute (Tianjin, China).

### Animal Preparation for Two-Photon Laser Scanning Microscopy Imaging

Prior to *in vivo* imaging, surgical preparation was performed as previously described (13, 15). Mice received 4.5% isoflurane and anesthesia and were maintained at 1%–1.5% isoflurane. Mice were positioned prone in a stereotactic frame. Body temperature was maintained at 37°C using a circulating warm-water blanket. After the skin is disinfected, an incision was made along the midline of the scalp. The periosteum was removed from the surface of skull to expose the bone suture mark. A cranial window with a diameter of 6 mm was created in the left and right frontoparietal cortex. During this procedure, hemostasis was performed in the soft tissue by monopolar cautery and Gelfoam strips in the bone. The skull and dura mater were removed to expose the brain. The surface of the brain was repeatedly rinsed to ensure no active bleeding. An 8 mm diameter removable cover glass was used to cover the craniotomy window and PBS was filled between the cover and brain. The cover glass was kept clean with sterile saline to facilitate two-photon microscopy.

### Two-Photon Laser Scanning Microscopy

A multi-photon laser scanning microscope (FVMPE-RS, Olympus, USA) attached to a laser generator with 680- to 1020-nm tuning range (Model: Chameleon coherent, UK) was controlled by FluoView software (FV31S, Olympus, USA). *In vivo* imaging was performed using 25X water-immersion objective lens (NA1.05, Olympus, USA). The vasculature was visualized by injection of Texas Red-dextran (MW: 70 kDa, 0.1 mL of 1% in saline; Invitrogen, USA) *via* the tail vein. The motion of red blood cells (RBCs) was detected with two-photon microscope and the cortical arterioles (diameter: 30-50 µm, subsurface depth: 10-20 µm) were imaged using Z scans (16). Repeated scans were performed along the central longitudinal axis of the selected vessel to determine flow velocity. The linear shadows formed by non-fluorescent particles in the vascular lumen was used to calculate the RBC velocity, which was proportional to the slope Δx/Δt. Thrombus was produced by laser irradiation as previously described (15). Localized vascular injury was induced by multiple pulse laser-irradiation on the arteriole (~30 x 50 µm ellipse field) until the thrombus formed. The emission wavelength was 920 nm. The thrombus occupied the entire lumen cross-section for approximately 6 min. Thrombosis was characterized by the dilatation of the irradiated segment, the emergence of bright fluorescence along the vessel wall, and a nonfluorescent mass within the irradiated arteriole. The laser pulse energy ranges from 0.1 to 0.6 mW, and intermittent scanning was used to avoid dye overflow and vascular rupture. Animals were not used if a blood vessel ruptures. The whole thrombus was scanned from top to bottom every 1 µm under two-photon microscope from 60 min after thrombosis to 90 min after vehicle or Ozanimod treatment. The thrombus volume was determined by 3D imaging and was determined by multiplying the sum of the thrombus area in a series of scanned slices by the section thickness (1 µm).

### Drug Administration

Animals were randomly assigned to tPA, ozanimod or vehicle by intravenous tail vein injection. Ozanimod (MCE, Monmouth, NJ) was dissolved in 5% DMSO + 0.9% saline as previously described and stored at 4°C (17). Mice were treated with Vehicle (100 µl), Vehicle (50 µl) + low-dose tPA (5 mg/kg, 50 µl), Ozanimod (0.6 mg/kg, 50 µl) + low-dose tPA (5 mg/kg, 50 µl) or standard dose tPA (10mg/kg, 100µl) at 60 min after vascular injury induced by laser irradiation. Experimental groups were as followed, A) Vehicle (100 µl), B) Vehicle (50 µl) + low-dose tPA (5 mg/kg, 50 µl), C) Ozanimod (0.6 mg/kg, 50 µl) + low-dose tPA (5 mg/kg, 50 µl), D) standard dose tPA (10mg/kg, 100µl). 

### Assessments of Bleeding Time, Coagulation Time

After anesthesia, the tails of the mouse were cut off by 2 mm with a scalpel. Then the truncated tail was immersed in a 15 ml transparent tube containing normal saline at 37°C. The observation period of bleeding time was 20 min. If no bleeding occurred within 30 s, the bleeding was considered stopped. This time was identified as the bleeding time. To assess coagulation time, angular venous blood from anesthetized animals were drawn into 1 mm diameter glass capillaries. The capillary was laid flat on the bench after being completely filled with blood. Then, a small section of the end of the capillary was snapped every 30 s until a fibrin thread appeared. Next, a small section of the end of the capillary tube was carefully snapped every 30 s until a fibrin thread appeared. Both bleeding time and coagulation time were measured at 90 min after treatment with Ozanimod (0.6 mg/kg, i.v.) low-dose tPA (5mg/kg, i.v.), Ozanimod (0.6mg/kg, i.v.) + low-dose tPA (5mg/kg, i.v.) or vehicle.

### Immunostaining

After anesthesia, the mice were perfused with 10 ml cold PBS and 10 ml 4% paraformaldehyde. Brain tissues were harvested and fixed in 4% paraformaldehyde overnight, and then dehydrated with 30% sucrose. The brain tissue was embedded in OCT, frozen, and sectioned (20 μm thickness). Brain sections were incubated for 30 min at room temperature in a blocking buffer (5% goat serum, 1% BSA, 0.3% Triton X-100), and then incubated with primary antibodies against mouse CD45 (1:200, 13917 Cell Signaling, Danvers, MA) or CD3 (10 µg/mL, MAB4841 R&D, Minneapolis, MN) at 4°C overnight. After the excess primary antibody was washed away with PBS, the brain sections were incubated overnight at 4°C with appropriate fluorochrome-conjugated secondary antibodies. DAPI (H-1200, Vector, Burlingame, CA) was used to counterstain cell nuclei the next day. Confocal Z stacks were acquired from the thrombus area labeled by Texas-Red-Dextran using a Zeiss 710 microscope with ×20 objective, NA 0.8 (Oberkochen, Germany). Data were analyzed with Image J software (National Institutes of Health, Washington, DC).

### Statistical Analysis

Randomization was performed using GraphPad QuickCalcs. Data were collected and analyzed by investigators that were blinded to experimental groups. A two-tailed unpaired Student’s t test was used to compare two independent groups. One-way analysis of variance (ANOVA) followed by Tukey *post-hoc* test was used for comparison of multiple groups. Statistical analysis was performed using Prism 8.0 software (GraphPad, San Diego, CA). Data are shown as mean ± SD. Values of p < 0.05 were considered significant.

## Results

### Effects of Subthrombolytic Dose of tPA on Microvascular Hemodynamics After Thrombosis

**Figure 1:**
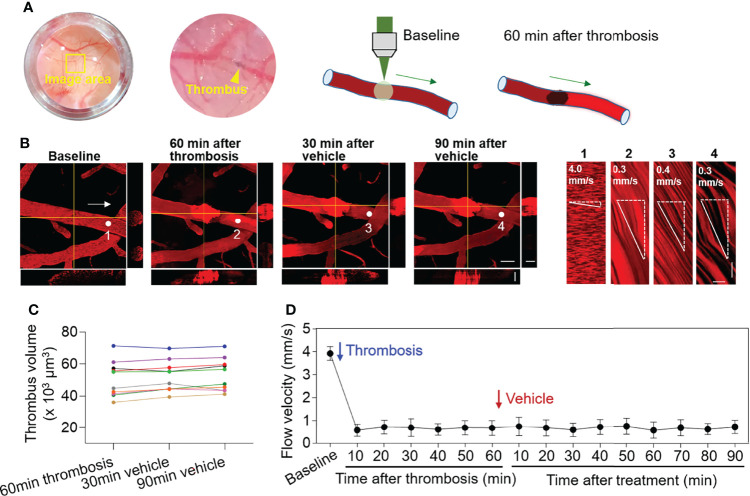
Effect of thrombosis on flow velocity in cortical arterioles measured by 2‐photon laser scanning microscopy. **(A)** Images and diagrams show the induction of thrombosis in cortical arterioles using 2‐photon laser scanning microscopy. **(B)** Z-stack Images show cortical arterioles at baseline, 60 min after thrombosis and up to 90 min after vehicle treatment. 3D images show horizontal and vertical views of thrombus. The yellow lines indicate the horizontal and vertical cross sections of thrombus. White arrows indicate the direction of blood flow. White dots and numbers indicate individual location to measure flow velocity. Scale bar: 50 μm in thrombus images, 30 μm in cross sections. Line scans were performed to measure flow velocity along the longitudinal axis of arterioles of interest (flow velocity is expressed as mm per second). The slopes (Δx/Δt) of the measurement-angles are proportional to flow velocity. Scale bar: 40 μm. Time bar: 0.1 sec. **(C)** Line graph shows thrombus volume at 60 min after thrombosis, 30 min and 90 min after vehicle treatment. **(D)** Flow velocity at indicated time points. n = 10 per group. Mean ± SD.

Laser irradiation was used to produce thrombosis in selected cortical arterioles (diameter: 30-50 µm) under two‐photon microscopy (**Figure 1A**). Laser irradiation-induced thrombus occupies the luminal space without vessel rupture or subsequent dye extravasation (**Figure 1B**). After thrombosis, flow velocity in selected cortical arterioles immediately declined (**Figure 1B**), and the volume of thrombus remained stable (p > 0.05, 60 min after thrombosis vs. vehicle treatment at indicated time points, **Figures 1B, C**). The reduction of flow velocity persisted and was not altered by vehicle treatment (p > 0.05, 60 min after thrombosis vs. vehicle treatment at indicated time points, **Figure 1D**).

**Figure 2:**
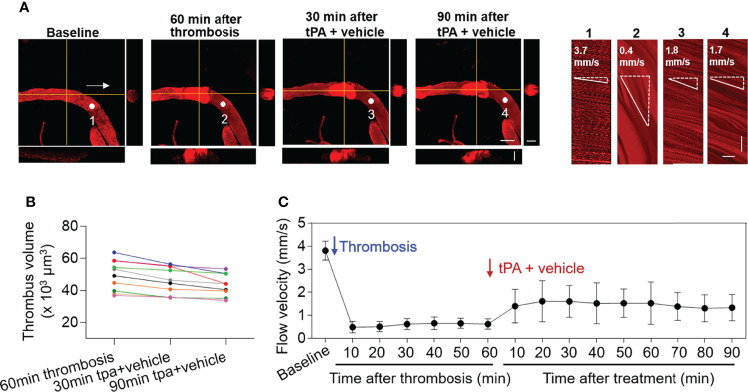
Effect of low-dose tPA on flow velocity in cortical arterioles following thrombosis. **(A)** Z-stack Images show cortical arterioles and flow velocity at baseline, 60 min after thrombosis and up to 90 min after vehicle plus low-dose tPA (5 mg/kg, i.v.) treatment. 3D images show horizontal and vertical views of thrombus. The yellow lines indicate the horizontal and vertical cross sections of thrombus. White arrows indicate the direction of blood flow. White dots and numbers indicate individual location to measure flow velocity. Scale bar: 50 μm in thrombus images, 30 μm in cross sections. Line scans were performed to measure flow velocity along the longitudinal axis of arterioles of interest (flow velocity is expressed as mm per second). The slopes (Δx/Δt) of the measurement-angles are proportional to flow velocity. Scale bar: 40 μm. Time bar: 0.1 sec. **(B)** Line graph shows thrombus volume at 60 min after thrombosis, 30 min and 90 min after tPA + vehicle treatment. **(C)** Flow velocity at indicated time points. n = 10 per group. Mean ± SD.

After treatment at 60 min of thrombosis, mice received a subthrombolytic dose of tPA (5 mg/kg, i.v. injection) along with vehicle. The volume of individual thrombus and flow velocity were monitored until 90 min after treatment (**Figure 2A**). We did not find significant alterations of thrombus volume (p > 0.05 vs. 60 min after thrombosis vs. 5 mg/kg tPA treatment at indicated time points) (**Figure 2B**). Flow velocity was increased following administration of low-dose tPA (5mg/kg) (**Figure 2C**).

### Selective S1PR1 Modulation Enhances Thrombolytic Effect of Low-Dose tPA

**Figure 3:**
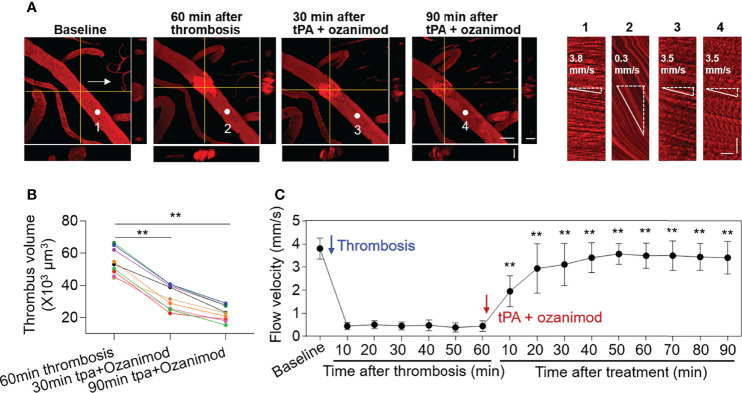
Effect of ozanimod and low-dose tPA treatment on flow velocity in cortical arterioles following thrombosis. **(A)** Z-stack Images show cortical arterioles and flow velocity at baseline, 60 min after thrombosis and up to 90 min after ozanimod (0.6 mg/kg, i.v.) plus low-dose tPA (5 mg/kg, i.v.) treatment. 3D images show horizontal and vertical views of thrombus. The yellow lines indicate the horizontal and vertical cross sections of thrombus. White arrows indicate the direction of blood flow. White dots and numbers indicate individual location to measure flow velocity. Scale bar: 50 μm in thrombus images, 30 μm in cross sections. Line scans were performed to measure flow velocity along the longitudinal axis of arterioles of interest (flow velocity is expressed as mm per second). The slopes (Δx/Δt) of the measurement-angles are proportional to flow velocity. Scale bar: 40 μm. Time bar: 0.1 sec. **(B)** Line graph shows thrombus volume at 60 min after thrombosis, 30 min and 90 min after tPA + Ozanimod treatment. **p<0.01 versus 60 min after thrombosis. **(C)** Flow velocity at indicated time points. n = 10 per group. Mean ± SD. **p<0.01 versus vehicle + tPA group.

To examine the impact of S1PR1 modulation on thrombolytic effect of low-dose tPA, we monitored thrombus volume and microvascular flow velocity in mice receiving a selective S1PR1 modulator ozanimod (0.6 mg/kg, i.v.) along with low-dose tPA (5 mg/kg, i.v.). The dose of ozanimod (0.6 mg/kg) was chosen because it is sufficient to induce lymphopenia as previously described (14). We found a notable reduction of thrombus volume and a dramatic increase of flow velocity in mice receiving ozanimod along with low-dose tPA (**Figures 3A–C**).

Notably, ozanimod alone also improved blood flow following thrombosis starting from 20 min after treatment (20 min: ozanimod vs. vehicle: 1.3 ± 0.6 mm/s vs. 0.7 ± 0.4 mm/s, p=0.018. 90 min: ozanimod vs. vehicle: 1.7 ± 0.6 mm/s vs. 0.7 ± 0.3 mm/s, p=0.012. Ozanimod: n = 6, vehicle: n = 10). Ozanimod-induced improvement of blood flow velocity was further augmented starting from 10 min after treatment in the presence of low-dose tPA (10 min: ozanimod vs. Ozanimod + 5mg/kg tPA: 0.7 ± 0.3 mm/s vs. 2.0 ± 0.7 mm/s, p<0.001; 90 min: ozanimod vs. ozanimod + 0.5mg/kg tPA: 1.7 ± 0.6 mm/s vs. 3.4 ± 0.7 mm/s, p<0.001. Ozanimod: n = 6, Ozanimod + 0.5mg/kg tPA: n = 10).

Additionally, we found that there is no significant difference between standard dose tPA and low-dose tPA + ozanimod until 90 min after treatment (10 min: 10 mg/kg tPA vs. ozanimod + 5 mg/kg tPA: 1.9 ± 0.8 vs. 2.0 ± 0.7, p=0.90; 90 min: 10 mg/kg tPA vs. ozanimod + 5 mg/kg tPA: 3.7 ± 0.4 vs. 3.4 ± 0.7, p=0.37. 10 mg/kg tPA: n=6, ozanimod + 0.5 mg/kg tPA: n=10). These results suggest that S1PR1 modulation enhances thrombolytic effect of low-dose tPA.

### Selective S1PR1 Modulation Does Not Affect Bleeding Time and Coagulation Time

**Figure 4:**
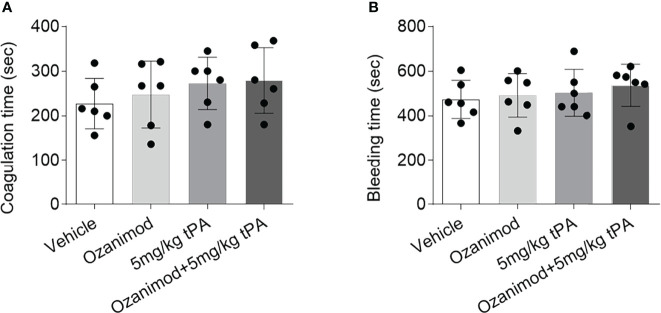
Effect of ozanimod on coagulation and bleeding time. **(A)** Bar graph shows coagulation time in mice receiving vehicle, ozanimod (0.6 mg/kg, i.v.), 5 mg/kg tPA or ozanimod (0.6 mg/kg) + 5 mg/kg tPA. **(B)** Bar graph shows bleeding time in mice receiving vehicle, ozanimod (0.6 mg/kg, i.v.), 5 mg/kg tPA or ozanimod (0.6 mg/kg) + 5 mg/kg tPA. n = 6 per group. Mean ± SD.

To test whether S1PR1 modulation improves thrombolytic effect of low-dose tPA involves its action on coagulation system, we measured bleeding time and coagulation time in mice receiving ozanimod or vehicle. We did not find a discernable impact of ozanimod on coagulation time and bleeding time (**Figures 4A, B**). Notably, low-dose tPA had minimal effect on bleeding time and coagulation time (**Figures 4A, B**). To test whether the beneficial effects of ozanimod on tPA thrombolysis involves the potential impact of ozanimod to amplify the effects of tPA on coagulation system, we also compared the bleeding time and coagulation time in groups of mice receiving low-dose tPA + vehicle or low-dose tPA + ozanimod. Similarly, we did not find significant alterations (**Figures 4A, B**).

### Selective S1PR1 Modulation Reduces Thrombus Volume and Lymphocytes Within Thrombus

**Figure 5:**
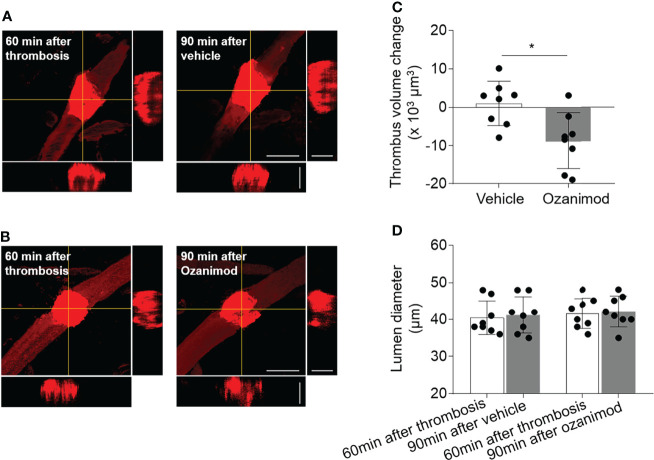
Effect of ozanimod on thrombus volume and lumen diameter in cortical arterioles following thrombosis. **(A, B)**. Z-stack Images show thrombus at 60 min after thrombosis and 90 min after ozanimod (0.6 mg/kg, i.v.) or vehicle treatment. 3D images show horizontal and vertical views of thrombus. The yellow lines indicate the horizontal and vertical cross sections of thrombus. Scale bar: 50 μm in thrombus images, 30 μm in cross sections. **(C)**. Bar graph shows the changes of thrombus volume at 90 min after ozanimod or vehicle treatment. Volume change = thrombus volume at 90 min after treatment - baseline volume at 30 min after thrombosis. n = 8 per group. **(D)**. Bar graph shows the changes of lumen diameter at 90 min after ozanimod or vehicle treatment. Lumen diameter was calculated at 60 min after thrombosis and 90 min after ozanimod or vehicle treatment. n = 8 per group. Mean ± SD. *p < 0.05.

To understand how ozanimod enhances the thrombolytic effect of low-dose tPA, we measured the thrombus volume before and after ozanimod treatment by generating sequential z‐stack images during *in vivo* two‐photon imaging (**Figures 5A, B**). We found that ozanimod reduced thrombus volume (**Figure 5C**), but did not affect lumen diameter (**Figure 5D**), suggesting that S1PR1 modulation may enhance the thrombolytic effect of low-dose tPA by reducing thrombus volume.

**Figure 6:**
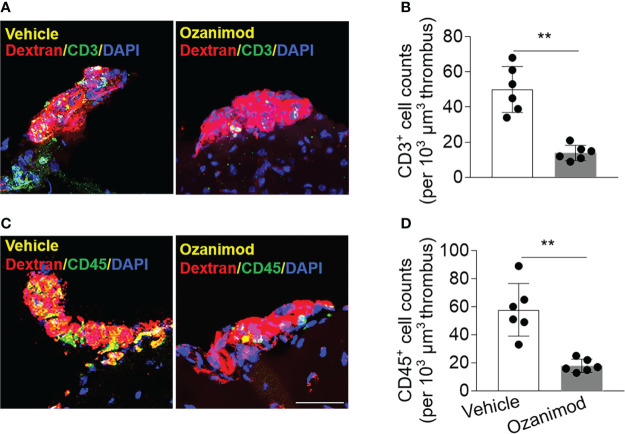
Effect of ozanimod on the composition of thrombus in cortical arterioles. Brain sections were prepared from brain tissues harvested at 90 min after ozanimod (0.6 mg/kg, i.v.) or vehicle treatment. **(A)** Z-stack immunostaining images show CD3^+^ T cells (green) within Dextran Texas-Red labeled thrombus in cortical arterioles. **(B)** Bar graph shows the counts of CD3^+^ T cells within thrombus. **(C)** Z-stack immunostaining images show CD45^+^ cells (green) within Dextran Texas-Red labeled thrombus. **(D)** Bar graph shows the counts of CD45^+^ cells (green) within thrombus. Scale bar: 40 μm. n = 6 per group. Mean ± SD. **p < 0.01.

As S1PR1 modulation is known to restrict the mobilization of peripheral lymphocytes, we examined whether ozanimod treatment could alter the composition of thrombus. To this end, we conducted immunostaining to examine the lymphocyte content within thrombus. Since T cells are a major subset of lymphocytes that contribute to thrombosis and respond to ozanimod treatment, we used T cell marker CD3 and leukocyte marker CD45. We found that ozanimod reduced the counts of CD3^+^ T cells and CD45^+^ cells within thrombus (**Figures 6A–D**). Ozanimod treatment also led to a reduction of neutrophils (Ly6G^+^ cells) and B cells (CD19^+^) within thrombus (Ly6G^+^ cells per 10^3^ µm thrombus: ozanimod vs. vehicle: 65.2 ± 27.4 vs. 143.5 ± 16.3, p<0.001, n=6 per group; CD19^+^ cells per 10^3^ µm thrombus: ozanimod vs. vehicle: 13.2 ± 3.2 vs. 22.7 ± 6.6, p=0.0097, n=6 per group). Similarly, we also found that ozanimod reduced the counts of CD3^+^ T cells and CD45^+^ cells within thrombus when used in combination with low-dose tPA (CD3^+^ cells per 10^3^ µm thrombus: ozanimod + 5 mg/kg tPA vs. vehicle + 5 mg/kg tPA: 21 ± 6.8 vs. 60.0 ± 20.1, p=0.013; CD45^+^ cells per 10^3^ µm thrombus: ozanimod + 5 mg/kg tPA vs. vehicle + 5 mg/kg tPA: 29.2 ± 7.5 vs. 80.7 ± 17.8, p<0.001; n = 6 per group). Together, these results suggest that S1PR1 modulation could modify thrombus composition following thrombosis, which may involve its effect on the restriction of T cell mobilization.

## Discussion

This two‐photon imaging study provides the novel evidence that selective S1PR1 modulation enhances the thrombolytic effect of low-dose tPA in cerebral arterioles after thrombosis. Based on real‐time visualization and quantitation of blood flow along with thrombus volume in cortical arterioles, we found that ozanimod, an FDA-approved drug to treat multiple sclerosis, can reduce thrombus volume and improve flow velocity when combined with subthrombolytic dose of tPA. These findings are in line with our findings in a previous clinical trial showing that S1PR modulation improves collateral flow as an adjunctive therapy to tPA thrombolysis (9). In addition, the antithrombotic activity of ozanimod and its effect to reduce the accumulation of T cells within thrombus suggest that lymphocytes are among the major targets for the benefit of ozanimod to improve thrombolytic effect of low-dose tPA.

Administration of tPA is associated with enhancement of lymphocyte egress, activation, and adhesion profiles in patients with ischemic stroke (18). In experimental stroke models, lymphocyte invasion into the neurovascular unit augments hemorrhagic transformation following tPA thrombolysis *via* propagation of neurovascular inflammation (18). Low-dose tPA reduces the risk of hemorrhagic events but were not more effective as compared to the standard dose (3, 4). Although the optimal dose of tPA remains to be further explored, AIS patients with a high risk of symptomatic intracranial hemorrhage might benefit from low-dose tPA (3, 4). In this respect, an adjunctive therapy is desirable to enhance the thrombolytic activity of low-dose tPA and maintain its safety advantage. The antithrombotic activity and effect to reduce T cells within thrombus place S1PR modulators as an ideal candidate for this purpose. Consistent with our recent study showing that a selective S1PR1 modulator RP101075, a metabolite of ozanimod, led to improved flow velocity following arteriole thrombosis induced by laser-irradiation (13), the present study has demonstrated that the addition of ozanimod to subthrombolytic dose of tPA results in a highly significant improvement of flow velocity at least until 90 min after treatment. Notably, ozanimod treatment reduced thrombus volume, suggesting that lymphocytes are a major target for the anti-thrombotic activity of ozanimod. This postulation is supported by our previous findings that the anti-thrombotic activity of S1PR1 modulator RP101075 was abolished in animals devoid of lymphocytes but not in animals receiving antibody depletion of monocytes and neutrophils (13).

Although tPA has been accepted as a standard of care for AIS patients, complete recanalization can only be achieved in less than one-third of patients receiving tPA thrombolysis (1, 19). Reocclusion occurs in approximately one-third of patients following initial recanalization and accounts for two-thirds of deterioration after initial improvement (1, 19). These observations suggest that clinical deterioration after successful thrombolysis is related to continued vascular occlusive events. As small vessels such as arterioles and capillaries are particularly vulnerable to injury after cerebral ischemia, microvascular endothelial cells may be targeted by inflammatory cells that initiate thrombosis and prevent reflow following tPA thrombolysis (5, 20). These considerations underscore the need for adjunctive therapies to enhance the benefit of thrombolysis. In the present study, we show that ozanimod can reduce the accumulation of T cells within the thrombus, suggesting the benefit of S1PR1 modulation to restrict inflammatory thrombosis and improve reflow following tPA thrombolysis. Additional measurements of T cells and B cells within the thrombus following full dose versus low-dose tPA treatment alone or with the combination of ozanimod are also necessary in the future to consolidate the involvement of blocking lymphocyte mobilization in the benefit of ozanimod.

The present results also provide strong support to our recent clinical trial showing that a S1PR modulator fingolimod enhances the efficacy of delayed tPA administration in AIS patients by promoting anterograde reperfusion and retrograde collateral flow (identifier: NCT02002390) (9). In that randomized, blinded endpoint trial, we enrolled 46 patients with internal carotid artery or middle cerebral artery proximal occlusion within 4.5 to 6 h from onset (9). The 23 patients receiving fingolimod plus tPA had better clinical improvement at 24 h and a favorable shift of mRS at day 90. Importantly, these 23 patients receiving fingolimod plus tPA exhibited a reduction in the perfusion lesion and suppressed infarct growth. This is accompanied by improved anterograde reperfusion of downstream territory and retrograde reperfusion from collateral circulation (9). Thus, these trial results suggest that S1PR modulation may enhance the efficacy of tPA thrombolysis. The present results lend further credence to such possibility. In this regard, larger clinical trials should be planned to assess the effects of S1PR modulators versus placebo in thrombolytic and nonthrombolytic cohorts. Results from this design may shed light on the postulation that S1PR modulation can enhance tPA thrombolysis.

In summary, our results demonstrate that the benefit of S1PR1 modulation enhance thrombolytic activity of low-dose tPA. These results will facilitate future design of larger clinical trials to determine the benefit of S1PR1 modulators as an adjunctive therapy to tPA thrombolysis.

## Data Availability Statement

All data generated in the current study are included in this published article. Source data supporting the findings of this manuscript are available from the authors upon reasonable request.

## Ethics Statement

The animal study was reviewed and approved by Animal Care and Use Committees of Tianjin Neurological Institute.

## Author Contributions

QL formulated the study concept. H-DL, RL, YK, WZ, CQ, DW, and HH performed experiments. H-DL, RL, and YK analyzed the data, interpreted the results, and assisted preparation of the manuscript. QL and HL wrote the paper. All authors contributed to the article and approved the submitted version.

## Funding

This study was supported in part by National Natural Science Foundation of China (82171284), National Natural Science Foundation of China (82101373), Natural Science Foundation of Tianjin Education Commission (2020KJ148) and Natural Science Foundation of Tianjin (18JCZDJC97600).

## Conflict of Interest

The authors declare that the research was conducted in the absence of any commercial or financial relationships that could be construed as a potential conflict of interest.

## Publisher’s Note

All claims expressed in this article are solely those of the authors and do not necessarily represent those of their affiliated organizations, or those of the publisher, the editors and the reviewers. Any product that may be evaluated in this article, or claim that may be made by its manufacturer, is not guaranteed or endorsed by the publisher.

## Abbreviations

S1PR1, sphingosine-1-phosphate receptor 1; tPA, tissue plasminogen activator; AIS, acute ischemic stroke; PBS, phosphate buffer saline; mRS, modified Rankin Scale.
